# Study protocol: MyoFit46—the cardiac sub-study of the MRC National Survey of Health and Development

**DOI:** 10.1186/s12872-022-02582-0

**Published:** 2022-04-01

**Authors:** Matthew Webber, Debbie Falconer, Mashael AlFarih, George Joy, Fiona Chan, Clare Davie, Lee Hamill Howes, Andrew Wong, Alicja Rapala, Anish Bhuva, Rhodri H. Davies, Christopher Morton, Jazmin Aguado-Sierra, Mariano Vazquez, Xuyuan Tao, Gunther Krausz, Slobodan Tanackovic, Christoph Guger, Hui Xue, Peter Kellman, Iain Pierce, Jonathan Schott, Rebecca Hardy, Nishi Chaturvedi, Yoram Rudy, James C. Moon, Pier D. Lambiase, Michele Orini, Alun D. Hughes, Gabriella Captur

**Affiliations:** 1 Barts Heart Centre, Barts Health NHS Trust, West Smithfield, London, ECIA 7BE UK; 2 Institute of Cardiovascular Science, University College London, Huntley Street, London, WC1E 6DD UK; 3 Centre for Inherited Heart Muscle Conditions, Department of Cardiology, Royal Free London NHS Foundation Trust, Pond Street, London, NW3 2QG UK; 4 Medical Research Council Unit for Lifelong Health and Ageing at UCL, 1-19 Torrington Place, London, WC1E 7HB UK; 5 Institute of Health Informatics, UCL, Euston Road, London, UK; 6 ELEM Biotech, S.L, Bristol, BS1 6QH UK; 7 Barcelona Supercomputing Center (BSC), 08034 Barcelona, Spain; 8 École Nationale Supérieure Des Arts Et Industries Textiles, 2 allée Louise et Victor Champier, 59056 Roubaix Cedex 1, France; 9 g.Tec Medical Engineering GmbH, Siernigtrabe 14, 4521 Schiedlberg, Austria; 10 National Heart, Lung, and Blood Institute, National Institutes of Health, Bethesda, MD 20892 USA; 11 Dementia Research Centre, UCL Queen Square Institute of Neurology, University College London, London, UK; 12 CLOSER, 55-59 Gordon Square, London, WC1H 0NU UK; 13 Cardiac Bioelectricity and Arrhythmia Center, Washington University, St. Louis, MO 63130 USA; 14 Department of Biomedical Engineering, Washington University, St. Louis, MO 63130 USA

**Keywords:** Subclinical myocardial dysfunction, Cardiovascular health, Life course risk factors, Risk trajectories, Cardiovascular magnetic resonance, Electrocardiographic imaging, Myocardial tissue characterization, Perfusion, 4-dimensional flow

## Abstract

**Background:**

The life course accumulation of overt and subclinical myocardial dysfunction contributes to older age mortality, frailty, disability and loss of independence. The Medical Research Council National Survey of Health and Development (NSHD) is the world’s longest running continued surveillance birth cohort providing a unique opportunity to understand life course determinants of myocardial dysfunction as part of MyoFit46–the cardiac sub-study of the NSHD.

**Methods:**

We aim to recruit 550 NSHD participants of approximately 75 years+ to undertake high-density surface electrocardiographic imaging (ECGI) and stress perfusion cardiovascular magnetic resonance (CMR). Through comprehensive myocardial tissue characterization and 4-dimensional flow we hope to better understand the burden of clinical and subclinical cardiovascular disease. Supercomputers will be used to combine the multi-scale ECGI and CMR datasets per participant. Rarely available, prospectively collected whole-of-life data on exposures, traditional risk factors and multimorbidity will be studied to identify risk trajectories, critical change periods, mediators and cumulative impacts on the myocardium.

**Discussion:**

By combining well curated, prospectively acquired longitudinal data of the NSHD with novel CMR–ECGI data and sharing these results and associated pipelines with the CMR community, MyoFit46 seeks to transform our understanding of how early, mid and later-life risk factor trajectories interact to determine the state of cardiovascular health in older age.

*Trial registration*: Prospectively registered on ClinicalTrials.gov with trial ID: 19/LO/1774 Multimorbidity Life-Course Approach to Myocardial Health- A Cardiac Sub-Study of the MCRC National Survey of Health and Development (NSHD).

## Background

Unprecedented numbers of people are now surviving into older age [1, 2] which means that the global trends for cardiovascular disease (CVD) prevalence will continue to increase, further adding to the burden of disease [3]. Improving our understanding of the causes of CVD is crucial to developing lifestyle or pharmacological interventions that can either prevent or delay the onset of disease.

The progressive structural, functional and hemodynamic changes affecting the myocardium as a result of ageing, lead to mortality, morbidity, disability and loss of independence in later life [4, 5]. Risk factors for cardiometabolic disease impact the heart from early childhood [6, 7] and with advancing age, the health of the myocardium is progressively compromised by overt stepwise and subclinical cumulative injury [5]. It is currently unclear how the age-dependent influences of multimorbidity, or the effect of early-life exposures, influence the various pathophysiological processes that lead to cardiac disease [8]. Prospectively collected data across the whole life course are required to address these research questions.

The Medical Research Council (MRC) National Survey of Health and Development (NSHD) is the longest running continuous study of human development in the world, which started as an English, Scottish and Welsh representative sample of 5,362 participants, born in one week in 1946 [9–12]. Repeated data collection waves since birth have provided detailed information on early-life exposures, traditional cardiometabolic risk factors and emergent multimorbidity [9, 11]. With members now aged ≥ 75, it is an opportune time to perform advanced cardiac phenotyping to understand which life course trajectories are conducive to the preservation of cardiovascular longevity. We propose to combine high spatial and temporal resolution electrocardiographic imaging (ECGI) and cardiovascular magnetic resonance (CMR) techniques to study the structure, function, electrophysiology (EP) and hemodynamics of the whole heart and correlate this information with the rich life course data that the NSHD cohort provides.

We describe here the research protocol of ‘MyoFit46’, a prospective longitudinal sub-study of 550 NSHD study members which is set to run for a total of 5 years. We summarize the study’s organization, funding, design, participant inclusion criteria, data sharing policy and analysis strategy.

## Methods

### Study organization

This prospective longitudinal NSHD sub-study will take place at the University College London (UCL) Bloomsbury Centre for Clinical Phenotyping in central London. MyoFit46 is funded by the British Heart Foundation (BHF) special project grant (to G.C. SP/20/2/34841) and supported by the MRC (MC UU 00019/1).

Separate ethical approval for the main NSHD study has been provided by Research Ethics Committees (REC) in England and Scotland as outlined in previous papers [9, 10, 13]. Ethical approval for this study was granted by the London Queen Square REC (REC: 19/LO/1774. London REC—Queen Square, IRAS: 254776). All participants will provide written informed consent to participate in the study and for their study data to be stored electronically in accordance with the Data Protection Act (2018) as laid out by the legal requirements of the General Data Protection Regulation (GDPR).

### Participants

Entry criteria to the study are based on maximizing the life course data available for analysis. We will recruit 550 NSHD participants, some of which have been recruited to other recent sub-studies [12] and who have not previously withdrawn, died, or remained untraced from the main study by age 70. Participants will have a rich life course data set available having participated in the majority of data collection sweeps (Table 1). The NSHD is a representative sample of 5362 males and females born in the UK in March 1946 originally intended to answer questions on fertility rates and obstetric servives [14]. At the 2014–2016 NSHD data sweep we obtained data on surviving study members that lived in Britain and with whom we still had contact [14], which has provided us with 2502 participants potentially available to invite for examination. The first 550 study members fulfilling these criteria and providing written consent will be included. Excluded individuals will be those with contraindications to contrast perfusion CMR, including but not limited to those with permanent implantable cardiac electronic devices, claustrophobia, renal failure, severe asthma or known trifascicular or higher degree conduction block on their resting surface 12-lead ECG.

**Table 1:** Summary of the various data collection sweeps so far undertaken on NSHD cohort participants over time

Data collections			Data collection year range (age, years)							
			1946 (birth)	1947–1950 (1–4 y)	1951–1960 (5–15 y)	1962–1977 (16–31 y)	1978–2003 (32–57 y)	2006–2010 (60–64 y)	2014–2015 (68–69 y)	2015-Present (65+)
			1	2	8	8	3	1	1	2+
Measurement	Social factors	Socioeconomic position	✓	✓	✓	✓	✓	✓	✓	✓
		Occupation	–	–	–	✓	✓	✓	✓	✓
		Education	–	–	✓	✓	–	–	–	–
	Health/physical measures	Morbidity and mortality	✓	✓	✓	✓	✓	✓	✓	✓
		Anthropometric measures	✓	✓	✓	✓	✓	✓	✓	✓
		Smoking status	–	–	–	✓	✓	✓	✓	✓
		Physical health	–	–	✓	–	✓	✓	✓	✓
		Diet	–	✓	–	–	✓	✓	✓	✓
		Respiratory function	–	–	–	–	✓	✓	✓	–
		Musculoskeletal function	–	–	–	–	✓	✓	✓	–
		Blood samples	–	–	–	–	✓	✓	✓	✓
		Urine samples	–	–	–	–	–	✓	–	✓
	Cardiovascular function	HR variability	–	–	–	–	–	✓	✓	✓
		12 lead ECG	–	–	–	–	–	✓	✓	✓
		Echocardiogram	–	–	–	–	–	✓	✓	✓
		Ambulatory blood pressure	–	–	–	–	–	✓	✓	✓
		Cardiac MRI	–	–	–	–	–	**–**	**–**	✓
	Neurological function	Brain MRI	–	–	–	–	–	**–**	✓	✓
		Cognitive function	–	–	✓	✓	✓	✓	✓	✓

Participants recruited to MyoFit46 may not necessarily have participated in all the data sweeps outlined in this table, but they will have participated in the majority of sweeps

ECG, Electrocardiogram; HR, heart rate; MRI, magnetic resonance imaging; NSHD, National Survey of Health and Development; y, years

### Study outline

**Fig. 1:**
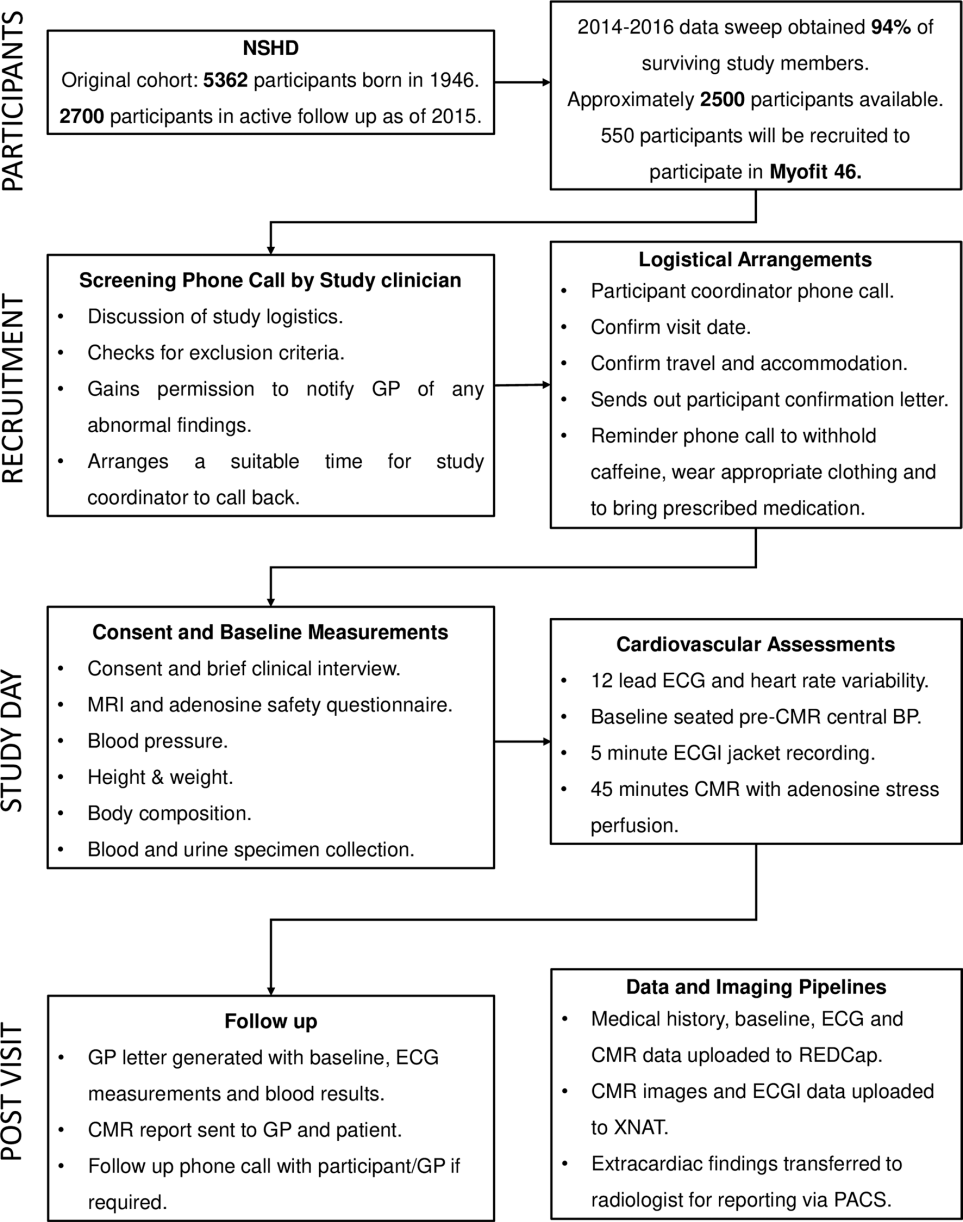
MyoFit46 study flowchart. BP, Blood pressure; CMR, cardiac magnetic resonance imaging; ECG, electrocardiogram; GP, general practitioner; MRI, magnetic resonance imaging; REDCap, research electronic data capture; PACS, picture archiving and communication system

All participants meeting the inclusion criteria will be invited to take part through an invitation letter and following verbal agreement, a suitable date/time for the CMR scan will be booked and transport arranged to and from the study center. In light of the severe acute respiratory syndrome coronavirus 2 (SARS-CoV-2) global pandemic specific measures will be implemented to minimize the risk of participant/investigator infection or transmission throughout the study period in accordance with changing government guidelines. A flowchart outlining the study protocol is shown in Fig. 1.

### Assessment and management of risk

A duty-of-care protocol based on the NSHD protocol used previously, in accordance with the MRC/Wellcome Trust guidelines [15] will be implemented for the purposes of feeding back health-related findings from the study. All baseline measurements will be routinely reported to the general practitioner with a copy also being sent to the participant.

All CMR images will be pre-reported by a cardiology clinical research fellow and finalized by a consultant cardiologist. Any extra-cardiac findings considered to be clinically significant will be secondarily reported by a consultant radiologist at University College London National Health Service (NHS) Foundation Trust. The study follows guidelines based on the UK Biobank imaging study [16] and CMR information is only reported back to the GP in the case that an abnormality is considered medically actionable. A non-exhaustive list of potential CMR findings considered reportable are summarized in Table 2.

**Table 2:** Overview of clinically significant reportable CMR findings

Category	Pathology
Ventricular structure	Moderate-severe LV hypertrophy
Ventricular function	Moderate-severe LV impairment
	Moderate-severe RV impairment
Atrial size	Moderate-severe atrial dilation
Valvular pathology	Structural valve abnormalities e.g. bicuspid aortic valve
	Moderate-severe valvular stenosis
	Moderate-severe valvular regurgitation
Pericardial effusion	Moderate-severe pericardial effusion
	Cardiac tamponade
Shunts	Ventricular septal defect
	Atrial septal defect
Cardiac masses	LV thrombus
	Intracardiac mass
	Valvular mass e.g. vegetation/fibroelastoma
Myocardial inflammation	Acute/chronic myocardial inflammation
Myocardial infarction	Acute/chronic myocardial infarction
Myocardial fibrosis	Clinically significant LGE
Perfusion defects	Clinically significant inducible perfusion defects
Extra cardiac findings	Other suspected, significant or life-threatening finding

LGE, Late gadolinium enhancement; LV, left ventricle; RV, right ventricle

All serious unexpected study-related adverse events will be reported immediately according to standard operating procedures for research studies carried out at UCL.

### Data handling and management

All data will be handled in accordance with the UK DPA 2018 as laid out by the UK GDPR. All clinical data will be pseudonymized by tokenization and will not bear the participant’s name or other directly identifiable data. The participant’s study identification number only, will be used for identification. All participant information including pre-screening questionnaires, relevant medical history, anthropometric measurements, baseline ECG and CMR data points will be imported into the secure research electronic data capture project (REDCap V.7.3.2) controlled by the principal investigator and accessible to named research assistants.

## Study protocol description

### Blood and urine specimen collection and storage

Blood samples will be collected and stored for future biomarker analysis. Two 4.0 ml ethylenediaminetetraacetic acid and two clotted 3.5 ml serum-separating tube whole blood samples will be collected. All tubes will be spun at 1300 g for 20 min at room temperature to generate supernatant (plasma), buffy coat and red blood cells which will be individually aliquoted into 1 ml, polypropylene, cryovials and stored at – 80 °C, for later analysis. One 10.5 ml PAXgene DNA tube and one 9.4 ml PAXgene RNA tube will be collected and stored upright for 24 h at – 20 °C, and then transferred to – 80 °C, for future analysis. Point-of-care testing will be carried out for creatinine using the StatSensor Xpress Creatinine monitor (Nova Biomedical, USA) and hemoglobin using HemoCue Hb 801 monitor (HemoCue AB, Sweden) that also permits derivation of hematocrit. A random urine sample will be collected by the participant in a polypropylene universal container and aliquoted into slender conical tubes for storage at – 80 °C.

### Baseline measurements

Height and weight will be measured according to standardized protocols previously described [12]. Bioimpedance body composition measurements (TANITA Cooperation, Japan) will also be recorded. Lying, sitting and 3-min standing blood pressure (BP) will be measured (OMROM MIT ELITE PLUS; OMRON Healthcare UK Ltd. Milton Keynes) on the left arm over 3 acquisitions. A resting 12-lead surface ECG will be recorded using a PC based ECG monitor (Cardioperfect Workstation; Welch Allyn, New York. USA). Resting heart rate (HR) variability will be assessed immediately after the standard ECG using the same lead positions for a total of 5 min.

### Electrocardiographic imaging (ECGI)

ECGI is the process of combining heart and torso geometry with multiple body surface potentials to generate epicardial electrograms and panoramic maps of cardiac excitation [17–19]. ECGI has been extensively validated in ex vivo animal studies using a torso-tank experimental method [20, 21] and in vivo animal experiments. It has also been shown to accurately correlate with invasive EP mapping in ventricular tachycardia (VT) [22], it has been validated when applied to cardiac computed tomography [23] and using a multi-electrode ECGI sock intraoperatively [24]. ECGI has also been successfully combined, by our group, with CMR in patients amyloidosis [25] and arrhythmogenic cardiomyopathy [26]. It is with this method that we can detect subtle EP abnormalities that are often missed by conventional 12-lead ECG [27]. The challenge for the cardiologist is identifying whether subtle EP abnormalities, which are more prevalent in the ageing myocardium, will put an individual at higher risk of sudden cardiac death or not [28]. In order to bridge this knowledge gap, ECGI will allow us to link regional EP aberrations with myocardial substrate changes for mechanistic insights into the aetiology of arrythmias. Our pipeline will enable reconstruction of epicardial surface biopotentials projected onto the cardiac geometry using inverse solution mathematics to measure epicardial activation and repolarization parameters which can then be spatially correlated with myocardial tissue characteristics derived by CMR. [29].

We recently developed a re-usable and dry-electrode-based CMR-compatible vest to permit high-throughput ECGI research and seamless integration with CMR (up to 3 Tesla [T]). This was achieved in collaboration with textile engineers at the École Nationale Supérieure des Arts et Industries Textiles (ENSAIT, Roubaix, France) and with g.Tec medical engineering GmbH (Schiedlberg, Austria). The technology consists of two matching wash-resilient and reusable garments: one embedded with 256 dry electrodes (the electrode vest) and the other (the CMR-safe marker vest) embedded with co-registered fiducial markers (Beekley medical, Bristol, Connecticut). The signal from each electrode is collected and amplified using the HIamp 256 bundle GT-8016/USBamp GT-0216 and processed for analysis in specially designed software, g.Recorder (g.Tec).

**Fig. 2:**
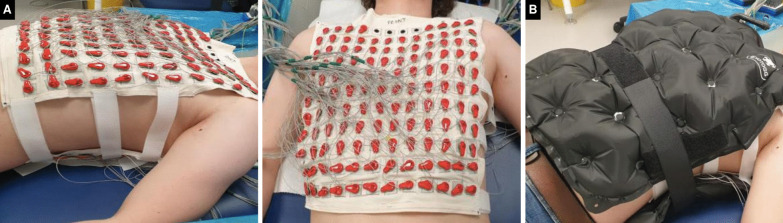
High-throughput reusable ECGI vest for CMR. **A** The garment is embedded with 256 uniformly distributed dry electrodes that connect to g.HIamp for the recordings. **B** To ensure good skin contact of the dry electrodes an inflatable gilet is worn over the electrode vest for the duration of the recordings. This design allows for rapid montage onto and off the chest. After the recording is complete the inflatable jacket and electrode vest are removed leaving only the marker vest that the participant then wears into the CMR scanner. ECGI, electrocardiographic imaging. Other abbreviations as in Fig. 1

Prior to CMR, the participant will wear the electrode vest which is secured onto the chest using adjustable fasteners. Body surface potentials will be measured from all electrodes at a sampling frequency of 2400 Hz for 5 min at rest, in the supine position. The electrode vest is then removed leaving only the marker vest which the participant wears inside the CMR scanner. Accurate electro-anatomical mapping of ECGI and CMR data will then be achieved through an initial 4 mm contiguous-slice transaxial black-blood thoracic stack for marker co-registration. Our ECGI workflow is summarized in Fig. 2.

### CMR

#### Outline

**Fig. 3:**
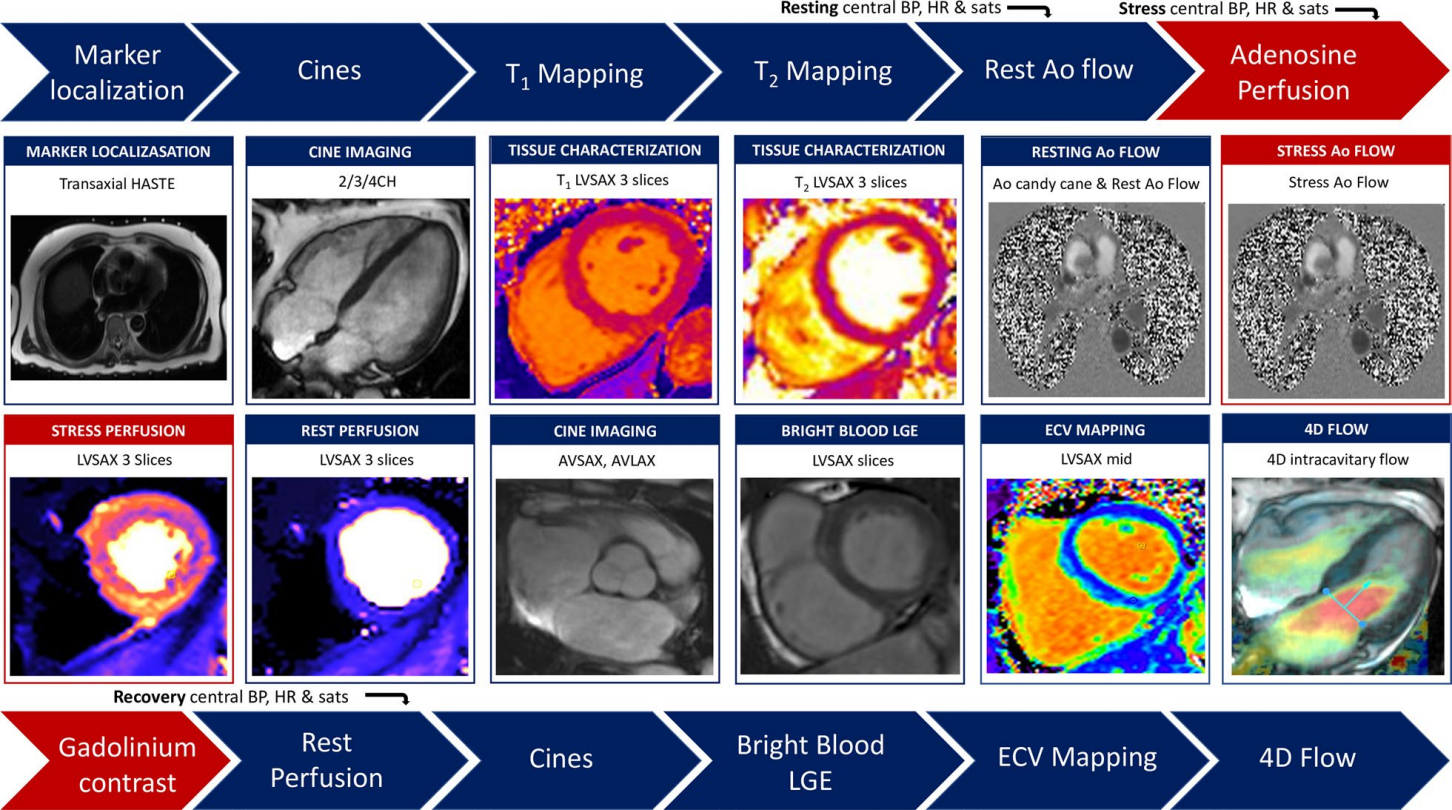
MyoFit46 CMR protocol. 2/3/4CH, 2/3/4 chamber; 4D, 4-dimensional; Ao, aorta; AVLAX, aortic valve long axis; AVSAX, aortic valve short axis; ECV, extracellular volume; HASTE, half-fourier single-shot turbo spin-echo; LGE, late gadolinium enhancement; LVLAX, left ventricular long axis; LVSAX, left ventricular short axis

Imaging will be performed on a 3 T [200 × 80mT/m/s x mT/m] MRI system (Magnetom Prisma, Serial Number 166032, Siemens, 60 cm bore) operating VE11C-SP01, with an 18-channel phased-array chest coil and spine array (up to 24-elements) equipped with Gadgetron [30] (Linux box, 24 cores). Participants will be scanned using a protocol which combines standard and advanced CMR imaging techniques and is designed to be completed within a 45 min scanning session. Standardized CMR protocols have been described in detail previously [31, 32]. Brachial and non-invasive central BP assessment using a Cardioscope II BP + device (USCOM, Sydney, Australia; cuff on the right arm) will be performed at baseline in the scanner bore, prior to the scan starting, followed by resting, peak stress (during adenosine administration) and recovery measurements, also inside the scanner bore. Continuous digital pulse oximeter monitoring will be obtained throughout using the Nonin 7500FO fiber optic MRI table-top pulse oximeter (Nonin, Plymouth, USA). The CMR protocol is outlined in Fig. 3 and MR sequence details summarized in Table 3.

**Table 3:** MyoFit46 CMR sequence parameters

Description	Transverse anatomy	LV short axis cine*	Native and post GBCA T_1_ map	Native T_2_ map	Aortic flow	Perfusion	LGE	4D flow
Pulse sequence	Dark blood TSE HASTE	Cine imaging bSSFP	bSSFP, MOCO, single shot MOLLI	bSSFP, MOCO single shot	Gradient echo phase contrast cine	bSSFP, MOCO, single shot	Bright blood MOCO bSSFP with PSIR	3D Spoiled gradient echo (WIP CS785B)
Flip angle (°)	160.0	50.0	20.0	70.0	20.0	50.0	50.0	15
TR/data acquisition window (ms)	251	29.1/–	–/167	–/148	9.24/–	144.0/70	–/203	39.76
TE (ms)	81.0/4.04	1.25/2.9	1.12/2.7	1.26/2.86	2.46/4.6	1.04/2.53	1.01/2.83	2.26/5.0
GRAPPA factor (parallel imaging)	2	2	2	2	2	3 (T-PAT)	2	7.6 (CS)
Slice gap (mm)	0.0	2.0	12.0	NA	NA	~ 100	2.0	0.0
Default field of View (mm)	490 × 398	380 × 285	360 × 270	360 × 270	380 × 214	360 × 270	360 × 270	400 × 310
Matrix size	256 × 135	256 × 140	256 × 144	192 × 120	192 × 97	192 × 111	256 × 144	160 × 102
Reconstructed voxel size	1.9 × 1.9 × 4.0	1.5 × 1.5 × 8.0	1.4 × 1.4 × 8.0	1.9 × 1.9 × 8.0	2.0 × 2.0 × 6.0	1.9 × 1.9 × 8.0	1.4 × 1.4 × 8.0	2.5 × 2.5 × 2.5
Calculated cardiac phases	1	30	1	1	1	1	1	20
ECG triggering/gating	PT	RG	PT	PT	RG	PT	PT	RG
Free breathing or breath hold	BH	BH	BH	BH	FB	FB	FB	FB (± navigated)
Orientation (slices) [PE direction]	Transverse (×110) [AP]	SA view base to apex (approximately × 12) [AP]	SA (×3 base, mid, apex) [AP]	Mid SA [AP]	Transverse at level of the PA bifurcation [AP]	SA (×3 base, mid, apex) [AP]	SA view base to apex (approximately × 9) [AP]	Sagittal [AP]

2-D, 2-dimensional; AP, antero-posterior; BH, breath hold; bSSFP, balanced steady-state free precession; CS, compressed sensing; FB, free breathing; GRAPPA, generalized auto-calibrating partially parallel acquisitions; HASTE, half-fourier single-shot turbo spin-echo; MOCO, motion-corrected; MOLLI, modified look-locker inversion recovery; PE, phase encoding; PSIR, phase sensitive inversion recovery; PT, prospectively triggered; RG, retrospectively gated; RL, right left; SA, short axis; TE, echo time; TPAT, temporal parallel imaging; TR, temporal resolution; TSE, turbo spin echo. Other abbreviations as in Table 2

*LV SAX cine stack is 8 mm slice thickness

#### Cardiac structure and function

Initial CMR scout imaging will include sagittal, coronal and transverse piloting of the chest in single heartbeat, free-breathing, acquisitions. This will be followed by a thin contiguously sliced transaxial set of turbo spin echo (TSE) images (4 mm thickness no gap) across the chest (approximately 90 slices) using a half-Fourier acquisition single-shot turbo spin echo (HASTE) sequence. These T_2_ weighted, dark blood, images will localize the fiducial markers on the ECGI vest and facilitate co-registration during post processing. Local 2^nd^ order cardiac shimming will be prescribed onto HASTE data and automatically copied to the rest of our protocolled sequences. To assess left ventricular (LV) structure and function we will acquire three long axis cines (vertical long axis, horizontal long axis and left ventricular outflow tract) and an LV short axis cine stack (SAX) using breath-held ECG retro-gated balanced steady state free precession (bSSFP) imaging. To assess right ventricular (RV) structure and function we will acquire RV outflow tract (sagittal and oblique-sagittal planes) and vertical RV long-axis bSSFP cines. For more detailed aortic valve (AV) assessment we will acquire coronal LV outflow tract (LVOT) and AV short axis bSSFP cines.

#### Tissue characterization

The native myocardial T_1_ time, before the administration of gadolinium based contrast agent (GBCA), can lengthen even in the absence of late gadolinium enhancement (LGE) and it is this ability to detect so-called ‘subclinical’ fibrosis that will make it particularly useful for our analysis of myocardial resilience in old age [33, 34]. In order to obtain native T_1_ maps we will acquire breath-held single shot bSSFP motion-corrected (MOCO) modified Look-Locker inversion recovery (MOLLI) images using prototype 5 s(3 s)3 s, at the basal, mid and apical LVSAX levels, with volume selective magnetic field strength (*B*_0_) shimming, in diastole [35, 36]. Standard deviation (SD) maps will be automatically generated and used to cross check the reliability of corresponding MOLLI T_1_ values in co-registered regions of interest, aiming for a SD of < 40 ms. A description of this technique has been outlined elsewhere [37].

To create extracellular volume (ECV) maps we will perform two co-registered T_1_ mapping acquisitions: before and approximately 10 min after infusion of the second dose of GBCA (Dotarem, Gadoterate meglumine, Guerbet, France) using MOLLI prototypes 5 s(3 s)3 s) and 4 s(1 s)3 s(1 s)2 s respectively, at the basal, mid and apical LVSAX slice. We will then generate pixel-wise ECV maps by determining the ratio of the change in T_1_ values, following correction for blood cell density (using validated point-of-care hematocrit readings) [31, 38, 39]. This will be done by transposing regions of interest (ROIs) in the septal midwall (or alternative remote myocardial zone) and blood pool from native onto the post-GBCA T_1_ maps. Two types of ECV maps will be generated per participant for cross-validation: (1) A synthetic ECV map, which will be generated directly from the scanner using a synthetic hematocrit derived from native T_1_ values, as described previously [39]; (2) A conventional ECV map, which will be generated at the end of each scan using the measured blood hematocrit (derived from the hemoglobin value obtained from point of care testing).

We will perform single-slice breath-held T_2_ mapping using a MOCO single shot bSSFP sequence at the level of the mid LV short axis and prior to GBCA administration.

#### Aortic flow imaging

**Fig. 4:**
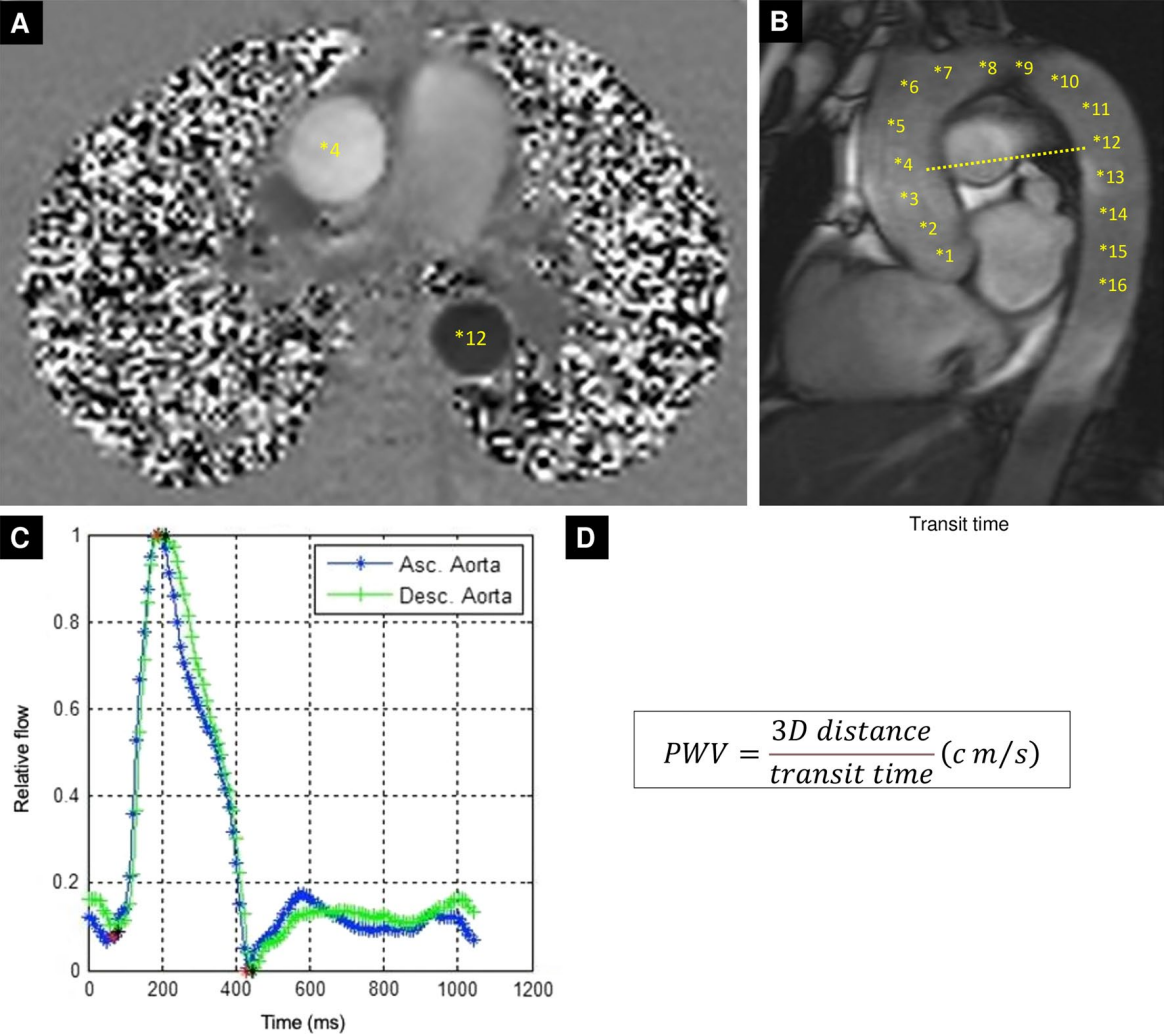
Measuring aortic pulse wave velocity (PWV). **A** Free breathing gradient echo phase contrast cine of the ascending aorta at the level of the pulmonary artery bifurcation. **B** Aortic ‘candy cane’ used to pilot the aortic flow. Points 4 and 12 are highlighted to show the linear plane used for transection and for measuring 3D distance using our dedicated software. **C** Flow wave curves of both the ascending and descending aorta after normalization for peak flow which are used to measure the transit time. **D** PWV (m/s) is then calculated as 3D distance divided by transit time. 3D, 3-dimensional; PWV, pulse wave velocity

With advancing age, biochemical, histological and blood flow changes in the main arteries result in wall stiffening [40]. Arterial stiffness has traditionally been measured in peripheral vessels due to ease of access, even though aortic stiffness is a stronger prognostic marker because of its resultant increase in LV afterload and wider downstream end-organ damage [41]. To analyze the effects of aortic stiffening in older age we will measure local aortic distensibility (derived from aortic area measurements) and regional pulse wave velocity (PWV) across the ascending aorta and aortic arch. Both regional and local measurements of aortic stiffness capture variation in aortic tissue composition across its course, but have different sensitivities to pathology related to arterial stiffening and are associated with different cardiovascular outcomes [42]. In order to obtain measurements of aortic length, we will initially acquire an ECG-gated, free-breathing sagittal cine of the aorta (‘candy cane’ view). This image will then serve as a localizer to pilot blood-flow velocity maps at the level of the bifurcation of the main pulmonary artery transecting the ascending and descending thoracic aorta. We will use a high temporal resolution phase-contrast free-breathing ECG-gated gradient echo sequence as previously described [31, 32, 43] (imaging parameters provided in Table 3). Aortic flow imaging will be performed at baseline and repeated during peak stress (immediately before the stress perfusion acquisition) alongside simultaneous pressure waveform and pulse oximetry measurements. An outline of the methods used to derive pulse wave velocity is summarized in Fig. 4. Measurements of flow velocity and pressure waveforms will be used to derive wave intensity, wave separation, reservoir pressure and reflection magnitude as previously described [44].

#### Stress perfusion imaging

For myocardial ischemia evaluation we will perform stress perfusion imaging using adenosine on all participants using a dual-cannula approach (typically sited in the antecubital fossae) which has been previously described [45]. Intravenous adenosine will be infused peripherally at 140 µg/kg/min from one of the cannulas for a minimum of 3 min and a maximum of 4 min to achieve an adequate response (assessed through a HR increment of 10 beats per minute and/or the development of symptoms). A higher dose of 175 µg/kg/min may be infused for a further 2 min in the event of limited symptoms or failure to increment HR by a minimum of 10 beats per minute. The first dose of Dotarem (0.05 mmol/kg) will be injected via the other canula at a rate of 4 mL/second along with 20 mL of saline. The adenosine infusion will be stopped during the first pass perfusion image acquisition which will be acquired using a free-breathing MOCO saturation-recovery bSSFP sequence to cover 3 LVSAX slices across every heartbeat. Approximately 10 min following peak stress, rest perfusion imaging (using the same sequences described above) will then be completed without adenosine, using the second dose of Dotarem.

#### Late gadolinium enhancement

To assess the presence of LGE we will acquire an LVSAX stack of conventional (‘bright-blood’) LGE images using a free-breathing MOCO bSSFP sequence with phase-sensitive inversion-recovery (PSIR) [46]. LGE acquisitions will commence approximately 5 min after the second dose of Dotarem.

#### 4-Dimensional flow

Four-dimensional (4-D) intracavitary flow CMR provides comprehensive hemodynamic flow assessments, taking into account the multidirectional and multidimensional nature of blood flow through the cardiac chambers [47] with the potential to improve our understanding of the ageing cardiovascular phenotype. We will compare the use of respiratory-navigated vs. free-breathing retrospectively ECG-gated, spoiled gradient echo sequences (work-in-progress CS785B, Siemens Healthineers, Erlangen, Germany), with field of view covering the LVOT, proximal aorta, and all 4 cardiac chambers. The acquisition will be performed at the end of the CMR protocol with GBCA on board to allow for improved blood myocardial contrast. Sequence parameters based on the published literature [48] are provided in Table 3.

#### Quality assurance

Normal values for key CMR biomarkers collected in MyoFit46 will be generated from healthy volunteer datasets acquired using the same sequences on the same magnet system. The upper and lower range of normal values will be defined as the mean ± 1.96 standard deviations from the normal data, according to standardized recommendations [38].

Prior to commencing native myocardial T_1_ mapping in MyoFit46, two quality assurance steps will be implemented.

**Fig. 5:**
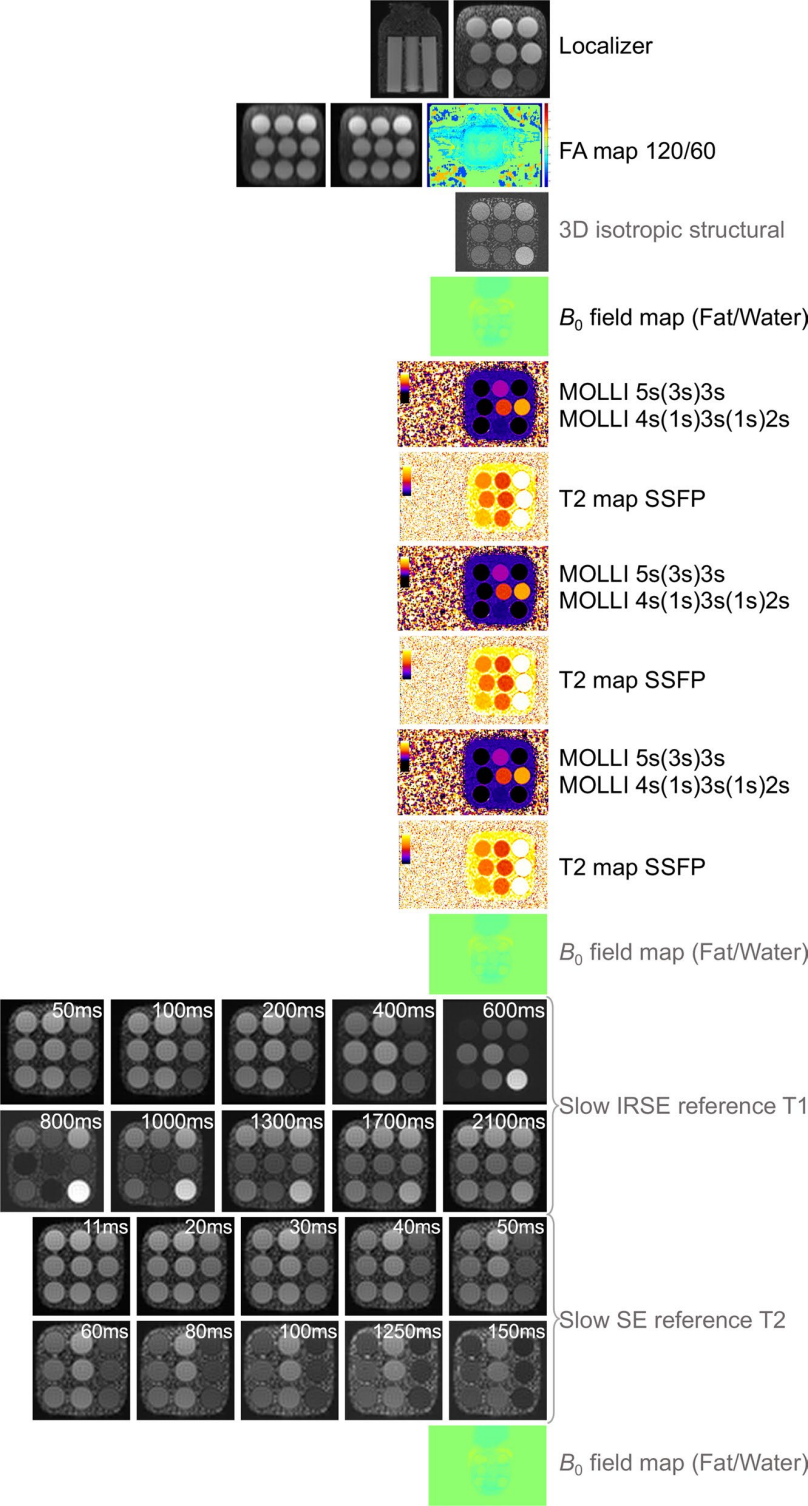
T_1_ and T_2_ Phantom QA framework. The same imaging protocol (defined here) is used for QA of multi-parametric T_1_ and T_2_ mapping data during the project lifecycle. Sequences in black are performed at baseline and then repeated every 8 weeks. Sequences in grey are performed at baseline and then for a total of 2 per year. 3D, 3-dimensional; FA, flip angle; IRSE, inversion recovery spin echo; MOLLI, modified Look-Locker inversion recovery; QA, quality assurance; SSFP, steady-state free precession

A quality assurance phantom testing protocol for T_1_, T_2_ and ECV mapping will be undertaken to verify the stability of our magnet-sequence combination over time and throughout the project lifecycle. We will use the medical-device grade T_1_ mapping and ECV standardization (T_1_MES) phantom [49] (field-strength specific for 3 T with regulatory clearance from the Food and Drug Administration and Conformité Européene-marking) as well as the T_2_ Phantom [50] (field-strength agnostic). Both devices have been previously developed by our group in collaboration with Resonance Health (Perth, Australia) and with the US and German National Metrology Institutes (National Institute of Standards and Technology and Physikalisch-Technische Bundesantalt respectively) [49, 51]. At study start and study close both phantoms (stored in the temperature-controlled MRI scanner environment) will be interrogated by flip angle maps and be scanned using slow overnight inversion recovery spin echo (IRSE) and multi-echo SE, to establish reference T_1_ and T_2_ values. At study start, study close and once every 2 months between these timepoints, the T_1_ MES phantom will additionally be scanned in accordance with the T_1_MES user manual [51, 52] using the fat/water sequence for field maps and using 3 repeats of the MOLLI 5 s(3 s)3 s and 4 s(1 s)3 s(1 s)2 s prototypes. At study start, study close and once every 2 months between these timepoints, the T_2_ phantom will additionally be scanned using the fat/water sequence and three repeats of the T_2_ mapping sequence. Phantom body temperature will be recorded prior to the start of each scan session, aiming for an ambient CMR room temperature of 21–22 °C. A schematic of our phantom-based quality assurance framework for MyoFit46 is presented in Fig. 5.

## Imaging pipelines and analyses

### Primary imaging analyses

#### Imaging pipelines

All CMR image DICOMs (Digital Imaging and Communications in Medicine), derived results and associated ROIs will be stored on a customized web-based server running XNAT 1.6.5 and hosted at UCL. DICOM-compliant imaging format will be used for all MRI data including raw list mode data and images that have been reconstructed on the scanner using the Gadgetron framework. CMR data will be analyzed using Circle Cardiovascular Imaging, cvi [42] version 5.3.2 (Calgary, Canada).

#### CMR analysis

Standardized CMR image interpretation and post-processing will be performed as previously described [31, 32, 53, 54]. Briefly, analyses will include volumetric measurements of the 4 cardiac chambers (using manual epicardial and endocardial contour tracings), assessment of biventricular and valvular function, analysis of native myocardial T_1_ and T_2_, stress perfusion imaging and LGE assessment. Papillary muscles and trabecular tissue will be consistently included in the LV mass measurements, as previously described [53, 54]. Left ventricular volumetric and maximal wall thickness analyses will additionally be performed using the previously validated openCARE artificial intelligence (AI) analysis platform. This platform has already been shown to have improved precision when compared to human observers in independent studies [55].

A standard operating procedure has been developed for the manual analysis of LGE, T_1_, T_2_ and ECV by trained researchers. For T_1_, T_2_ and ECV analysis, epicardial and endocardial contours will be drawn manually with 10% erosion from the blood myocardial boundary and divided according to the 16-segment American Heart Association (AHA) model. On the native and post-GBCA MOLLI T_1_ maps, a ROI will be drawn in the mid septum and co-registered with automatically generated pixel-wise SD maps. Semiautomated LGE quantification will be carried out using a 2 SD and a 5 SD approach following manual selection of an ROI in an area of remote/dark myocardium [36, 56]. We will also use the full width half maximum technique [57] for comparison against the other methods. Segments with LGE will be annotated to allow for subsequent interpretation of the mapping data [54].

#### Aortic flow analysis

Proximal ascending and descending thoracic aortic contours will be traced semi-automatically using validated software (ArtFun, INSERM U678, Paris, France) to derive flow-velocity waveforms [43, 58]. Aortic distensibility will be measured at local, specified regions of the aorta and calculated according to established methods [43, 59]. PWV over the upper thoracic aorta will be measured using length and transit time (from derived aortic velocity profiles) [43].

#### 4-Dimensional flow analysis

Intracavitary 4-D flow CMR will allow us to derive parameters such as turbulent kinetic energy, wall shear stress, PWV and variations in LV vortices [47, 60]. For example, previous studies have shown that there is an inverse relationship between LV diastolic vortices [61] and ageing and that turbulent kinetic energy could be a promising subclinical marker of LV dysfunction [62]. 4-D flow CMR also allows for detailed valve quantification with particularly promising results in mitral regurgitation, since it allows for direct quantification of flow at the level of the valve which remains valid even in the presence of other valvular lesions or shunts [63]. These markers of intracavitary hemodynamics will be used to characterize pathophysiological responses to ageing beyond conventional methods of flow. 4-D flow image post-processing will be performed using CAAS (PIE Medical Imaging, Maastricht, The Netherlands). During analysis we will ensure that any contours drawn in the magnitude images are cross-referenced with velocity images to take into account large movements and the effects of partial volume. Velocity data will be encoded into individual vectors, allowing us to assess local patterns of flow such as vortices, visualize and quantify wall shear stress and study kinetic energy [64].

#### ECGI analysis

**Fig. 6:**
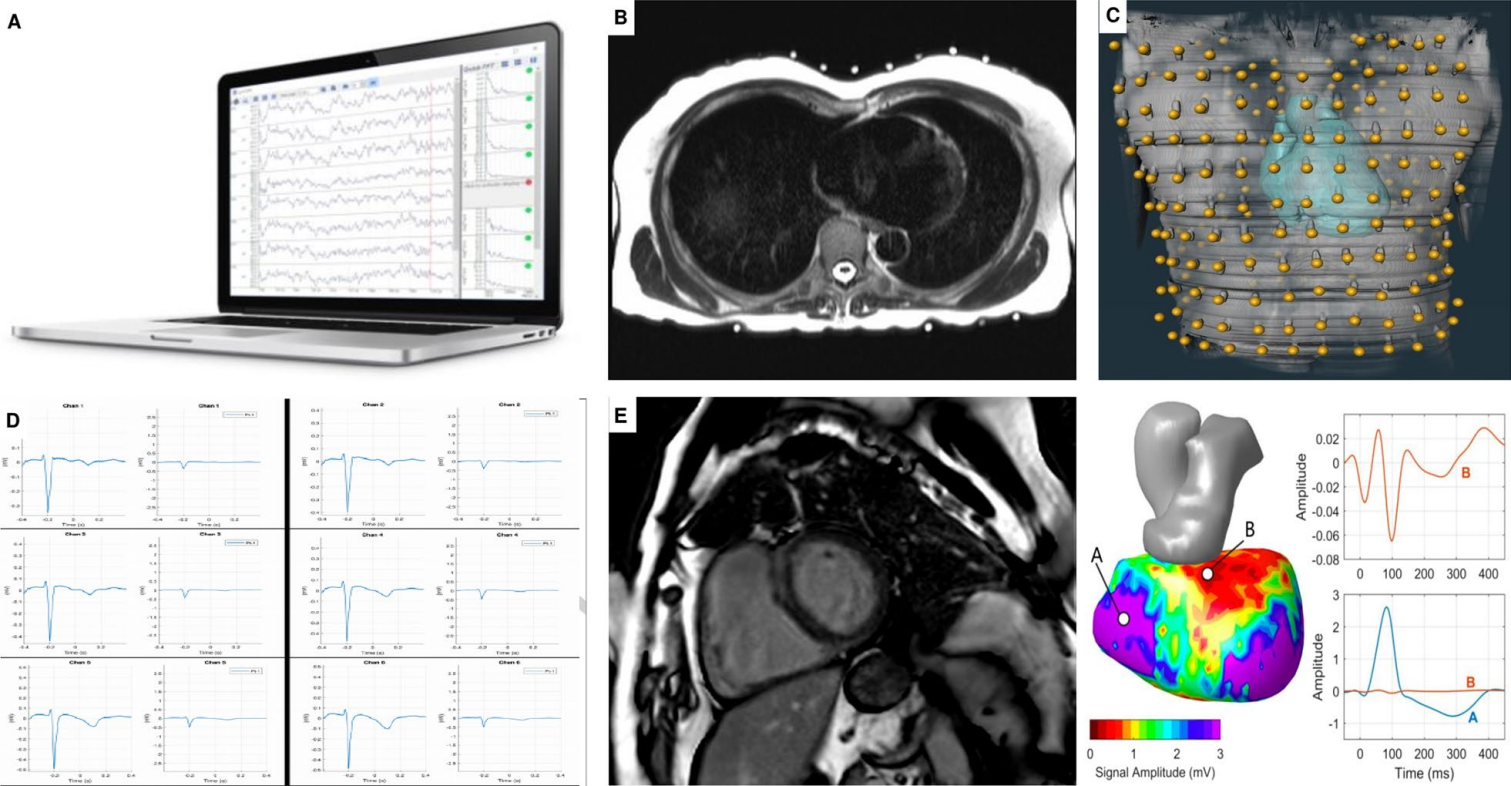
ECGI analysis workflow. **A** ECGI data is collected by g.Recorder software. **B** CMR image showing one transverse slice of the HASTE used to segment the epicardium. Note the fiducial MRI markers (white) situated on the anterior and posterior torso. **C** Ventricular segmentation of the same participant’s HASTE data, showing heart torso geometry and markers (Beekley medical, Bristol, Connecticut) following volume rendering, landmark localization and segmentation in Amira software (ThermoFisher, MA, USA, version 2020.3). **D** Signal averaging is performed using in house, customized software (Matlab, MathWorks, Natick, MA) and unipolar electrograms are then combined with Amira software heart torso geometry by solving the inverse problem of ECGI in collaboration with the Rudi laboratory. **E** Activation time isochrone maps in disease highlighting an area of fractionation corresponding to an area of LGE/scar in the mid anteroseptum of the left ventricle. EGM, electrogram. Other abbreviations as in Figs. 1, 2 and 3

Body surface ECGs collected by g.Recorder will be stored and analyzed offline using previously established methods [18, 25, 26]. Signal averaging will be performed using in-house, customized, software (Matlab, MathWorks, Natick, MA) to enhance the quality of the signals from body surface potentials. The location of the fiducial markers on the torso corresponding to the electrode positions will be combined with the heart-torso geometry obtained from the CMR HASTE stack and reconstructed to create epicardial meshes using commercially available software (Amira, ThermoFisher, MA, USA, version 2020.3). Unipolar epicardial electrograms will then be reconstructed by solving the inverse problem in collaboration with the Rudy laboratory [19, 26]. Generated epicardial maps will enable calculation of epicardial activation and repolarization times and corresponding isochrone maps will be generated for visualization. These maps will be used to identify discrete areas of abnormal electrophysiology and correlated with matching substrate abnormalities detected by CMR. An exemplar pilot isochrone map that we have created using this CMR-ECGI pipeline is reproduced in Fig. 6. At scale validation of the co-registration process with CMR has not been performed before and in order to address this we will carry out test–retest reproducibility and intra-/inter-observer variability studies. All raw and post-processed ECGI data will be stored securely in the project-specific UCL XNAT repository.

#### Cardiac computational modelling

In order to extend our multi-dimensional deep phenotyping of the ageing heart we will perform advanced cardiac computational modelling in collaboration with the Barcelona Supercomputing center (Barcelona, Spain). Using the established ‘Alya Red’ [65] supercomputer driven data modelling protocol [65–67], we will combine epicardial electrophysiological data (from ECGI) with the rich myocardial structural, functional, perfusion and flow data obtained from CMR to create personalized electro-mechanical-flow whole-heart simulations per participant [68].

### Study analysis Plan

#### Statistical analysis

Both overt and subclinical cardiovascular disease biomarkers (binary or continuous variables, such as LV ejection fraction, native T_1_, ECV, LGE, etc.) will be determined through this proposed CMR-ECGI deep phenotyping protocol and such biomarkers will serve as either outcome or predictor variables in the various analyses planned. On the one hand, MyoFit46 will unravel how key exposures during early-, mid- and later life (e.g. environmental pollution, communicable disease exposure, socioeconomic status, etc.) interact with traditional cardiovascular risk factors (e.g. smoking, obesity, alcohol intake, etc.) and multimorbidity to determine cardiovascular disease (outcome) in older age. On the other hand, we will also prospectively study the association between discovered cardiovascular disease biomarkers (predictors) and key outcomes of interest ascertained during long-term follow-up of the birth cohort participants (e.g. functional status or major adverse cardiovascular events such as hospital admissions, nonfatal stroke, myocardial infarction, cardiovascular death, etc.).

Since several of our exposures of interest are either categorical or represent trajectories/time-varying exposures; associations between exposures and outcomes will be analyzed using a range of statistical approaches relevant for life course data [69–71]. Associations between trajectories of exposures and cardiovascular disease biomarkers will be analyzed using latent growth curve analysis [71]. In order to subsequently link with outcomes we will use a two-step process as previously described [72]. We will use structural equation models using full information maximum likelihood to account for missing data in NSHD under missing at random assumptions.

Sample size estimates have been calculated to permit detection of a small effect size (Cohen’s *f*^2^ = 0.03) using multivariable regression analysis. A sample size of 533 achieves 80% power to detect an *f*^2^ ≥ 0.030 attributable to up to 9 independent variable(s) using an *F*-Test with a significance level (alpha) of 0.050. For categorical outcomes a logistic regression of a binary response variable on a continuous, normally distributed variable with a sample size of 533 observations achieves 92% power at a 0.05 significance level to detect a change corresponding to an odds ratio of 1.5.

### Data sharing

A data sharing policy will be published and managed through MRC Slylark website (https://skylark.ucl.ac.uk/Skylark) including access to research publications, talks presented at international conferences and links to academic social media accounts. Raw data will be archived for a minimum of 10 years within the UCL open XNAT server (https://ucl-open-xnat.cs.ucl.ac.uk/app/template/Login.vm#!) and these data will be shared with *bona fide* researchers along with the relevant clinical and demographic metadata that will be archived in REDCap. CMR and ECGI standard operating procedures will be electronically archived alongside the data, and as hard copies. Software pipelines will be stored in electronic lab books, including software version numbers.

## Discussion

MyoFit46 aims to combine the rich life course data of the NSHD with cutting-edge CMR and ECGI technologies and multi-dimensional cardiac simulations to provide new insights into the long-term myocardial sequelae of key exposures from birth. Uniquely the NSHD provides us with detailed, prospectively collected data since birth, allowing us to study the individual, combined and cumulative impacts of risk factors, and their mediators on myocardial health by older age. Previous older age cohorts have carried out CMR imaging, but none have performed CMR-ECGI integration with stress perfusion, multi-parametric mapping, and 4-D intracardiac flow as is being proposed here. ECGI will enable us to correlate overt and subclinical markers of arrhythmogenesis with CMR-derived structural and functional abnormalities. By merging these complex structural, functional, hemodynamic and electrophysiological data into cardiac computational models we be deepening our understanding of the ageing myocardium, representing a world first in terms of CMR-driven personalized care. We expect our results to impact public health by identifying novel risk factors for cardiovascular disease and addressing the growing challenge of multi-morbidity with the aim of maintaining myocardial health into older age for the benefit of humanity globally.

## Data Availability

Not applicable.

## Abbreviations

AHA: American Heart Association
AI: Artificial intelligence
AV: Aortic valve
BHF: British Heart Foundation
BP: Blood pressure
bSSFP: Balanced steady state free precession
CMR: Cardiovascular magnetic resonance
COVID-19: Coronavirus disease 2019
CVD: Cardiovascular disease
DICOM: Digital Imaging and Communications in Medicine
DPA: Data protection act
ECG: Electrocardiogram
ECGI: Electrocardiographic imaging
ECV: Extracellular volume
EP: Electrophysiology
FSE: Fast spin echo
4-D: Four-dimensional
GBCA: Gadolinium based contrast agent
GDPR: General Data Protection Regulation
HASTE: Half Fourier acquisition single shot turbo spin echo
HR: Heart rate
IRSE: Inversion recovery spin echo
LGE: Late gadolinium enhancement
LV: Left ventricle
LVOT: Left ventricular outflow tract
MOCO: Motion corrected
MOLLI: Modified look locker inversion recovery
MRC: Medical Research Council
MRI: Magnetic resonance imaging
NHS: National Health Service
NSHD: National Survey of Health and Development
PSIR: Phase-sensitive inversion-recovery
PWV: Pulse wave velocity
REC: Research Ethics Committee
REDCap: Research electronic data capture project
ROIs: Regions of interest
RV: Right ventricle
SARS-CoV-2: Severe acute respiratory distress coronavirus 2
SAX: Short axis stack
SD: Standard deviation
T: Tesla
TSE: Turbo spin echo
UCL: University College London
VT: Ventricular tachycardia

## Declarations

### Study status

This study is ongoing having commenced participant recruitment.
